# Technological and microbiological characterization of an industrial soft-sliced bread enriched with chitosan and its prebiotic activity

**DOI:** 10.1016/j.crfs.2024.100935

**Published:** 2024-11-28

**Authors:** Margherita D'Alessandro, Maria Alessia Schouten, Davide Gottardi, Sara Cortesi, Santina Romani, Francesca Patrignani

**Affiliations:** a Interdepartmental Centre for Industrial Agri-Food Research (CIRI-Agro), University of Bologna, Via Quinto Bucci 336, 47521, Cesena, Italy; b Department of Agricultural and Food Sciences (DISTAL), University of Bologna, Piazza Goidanich 60, 47521, Cesena, Italy; c Orva Spa, Via M. Tarroni 15, 48012, Bagnacavallo, RA, Italy

**Keywords:** Chitosan bread, Microbiology, Technological properties, Aroma compounds, Prebiotic activity, Industrial processability

## Abstract

Several studies have described the effects of chitosan as an ingredient in bread, particularly from a technological and functional point of view. However, these studies mainly focus on breads produced at lab scale with a short shelf life, which may not reflect the changes occurring in industrial production. Our study investigated the potential of using chitosan at an industrial scale to produce soft white bread, evaluating its impact on the final product's shelf life and providing deeper insights into the practical possibilities and limitations of its scalability. In particular, the rheological properties of the dough and the overall qualitative characteristics of the breads were evaluated when chitosan was used at 0.75 and 1.5%. The use of chitosan in bread dough increased its viscoelasticity, firmness and extensibility, making the dough more elastic but harder to mold and process industrially (extension resistance: 41.70 for 1.5% chitosan vs 22.55 for the control). Chitosan breads exhibited higher pH, aw (1.5%: 0.955 vs control: 0.934), firmness and a larger pore size, with a lower cut height and a more pronounced colour due to increased Maillard reactions. Microbiologically, the chitosan breads were within acceptable limits (<4 and 3 log CFU/g for aerobic mesophilic bacteria and yeasts, respectively) but showed no effect on spoilage microbiota. However, the addition of chitosan increased the prebiotic activity of the bread, as assessed by its ability to promote the growth of selected probiotics in simulated intestinal fluid, which has the potential to positively impact consumers' gut health.

## Highlights

• No studies explored chitosan's potential and limitations in industrial bread production.
• Chitosan increases bread dough's viscoelasticity, firmness, and elasticity, but hinders molding.
• Chitosan bread exhibited higher pH, aw, firmness, larger pore size and more pronounced color.
• Chitosan bread met microbiological limits but failed to control spoilage microbes.
• Chitosan addition increased prebiotic activity and enhanced flavor compounds in soft white bread.

## Graphical abstract

**Figure:**
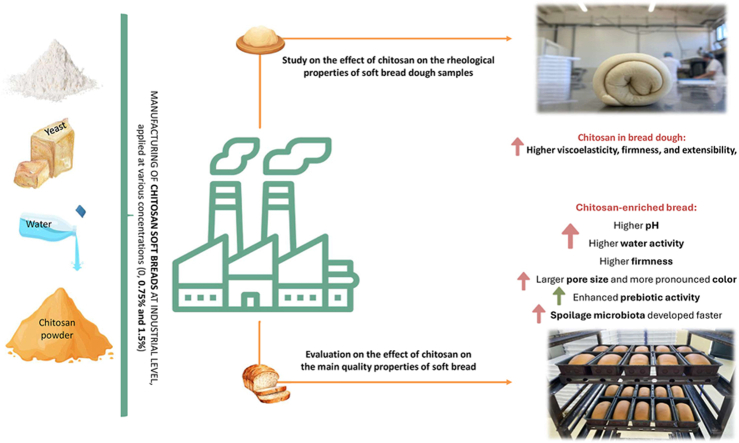

## 1 Introduction

Bread is one of the most consumed products in the world and is generally considered a very convenient food (Marinopoulou et al., 2020). Increasing bread consumption was reported as a 3.66% Compound Annual Growth Rate (CAGR) between 2020 and 2030 in the global market attributed to its convenience, nutritional value, and appealing taste (Custom Market Insights, 2023). Notably, among the different types of bread (i.e. whole grain bread, white bread, sliced or wrapped bread), soft white bread is one of the most consumed worldwide. According to Rahman et al. (2022), soft white bread now surpasses pasta, pseudocereals, rice and tubers in terms of consumption. Given the widespread consumption of soft white bread by consumers, this product is of great interest to the industry, especially in terms of new challenges and existing limitations that need to be addressed to improve the quality of this type of product. Regarding these limitations, soft white bread, like most types of bread, is highly susceptible to microbial spoilage, leading to unacceptable physiological, microbial and biochemical changes (Soltani et al., 2023). In fact, bread and other bakery products are among the most wasted foods and can account for up to 30% of total waste (Brancoli et al., 2019), with a significant proportion of waste due to microbial spoilage (Rahman et al., 2022). As suggested by Rahman et al. (2022), Novozyme, which surveyed over 4000 bread consumers across Europe, found that the main reason for discarding bread is mold. Therefore, bread spoilage represents a significant economic loss for both consumers and producers. In recent decades, the bakery industry has been trying to find alternative treatments and methods to extend bread shelf life and enhance its safety by increasingly applying natural preservatives and sustainable solutions. For instance, using microbial fermentation, particularly with lactic acid bacteria (Guimarães et al., 2018; Sadeghi et al., 2019; Zhang et al., 2019), biopreservatives such as essential oils (Debonne et al., 2018; Teodoro et al., 2014) or plant derivatives (Rizzello et al., 2015), along with the adoption of nanofibers (Fonseca et al., 2021), active packaging and modified atmospheric packaging (Qian et al., 2021). Moreover, fortifying cereal-based products with fiber-rich ingredients seems to be a promising approach for enhancing the dietary fiber intake. Dietary fibers have been part of human diet for centuries and have been renowned as sources of health-beneficial components without providing significant calories. Their consumption offers a multitude of health benefits, including protection against cardiovascular diseases, cancer, reduction of blood serum cholesterol, regulation of blood glucose levels, prevention of constipation, hemorrhoids, and diverticulitis (Ciudad-Mulero et al., 2019). One natural fiber with promising characteristics is chitosan, a linear polyamino-saccharide obtained through alkaline deacetylation of chitin, an organic polymer found naturally in the exoskeletons of crustaceans such as crabs, shrimp, and lobsters, as well as in marine zooplankton species like jellyfish and coral, and in the cell walls of yeast, mushrooms, and fungi (Nawawi et al., 2019; Shahidi and Abuzaytoun, 2005). Chitosan extracted from crustacean waste has received significant attention for addressing one of the primary environmental concerns within the food processing industry (Seenuvasan et al., 2020). In fact, due to its chemical structure, chitosan possesses antioxidant, antimicrobial (against bacteria such as *Salmonella*, *Listeria*, fungi and yeast), and emulsifying properties, as well as prebiotic, and health promoting biological activities (anti-hypercholesterolemic, anti-hypertensive, and anti-inflammatory) which can make it a valuable food ingredient for the development of innovative products (Zhou et al., 2021). Although the most promising applications of chitosan in food involve its use as edible film coating (Ghoshal and Singh, 2024) or encapsulating agents (Maleki et al., 2022) to extend the shelf life of food or enhance its functionality, chitosan-enriched breads have already been manufactured and evaluated for their properties. In fact, chitosan could be a remarkable ingredient for bread compared to conventional preservatives. This is due to its effective antimicrobial properties, its biodegradability, its potential health benefits and its natural origin, all of which respond to consumers' desire for healthier, simpler and more sustainable ingredients. According to Ghoshal and Mehta (2019) the addition of chitosan to bakery products could offer the opportunity to combine its beneficial technological properties with its health-promoting characteristics. Ribeiro et al. (2019) described the formation of an interlocked supramolecular assembly between gluten and chitosan which can potentially mitigate the celiac-toxicity of wheat flour. Moreover, Lafarga et al. (2013) and Rakkhumkaew and Pengsuk (2018) reported that the addition of chitosan to white bread inhibited *Bacillus cereus* and *Rhizopus* spp. growth and delayed the natural growth of moulds. However, these studies primarily relate to breads produced on a lab scale, with a short shelf life, which may not reflect the changes that occur when scaling up to an industrial production level. In addition, the antimicrobial properties of these breads have typically been evaluated through challenge test with target microorganisms. While this is a useful model, it may differ from a true shelf-life study. In this context, our research takes a step further by directly assessing the potential of chitosan in industrial bread production and its impact on shelf life. This study, conducted in collaboration with the food company Orva, aimed to evaluate the effects of chitosan at different concentrations (0.75% and 1.5%) in the formulation of soft white bread produced on an industrial scale and its impact on the overall qualitative properties. In particular, the rheological properties of bread doughs were analysed before and after proofing. Subsequently, the baked bread loaves were examined over a 60-day shelf-life, as proposed by the company involved, to evaluate the effects of chitosan on texture and color characteristics, microbiological quality, prebiotic capacity and volatile molecule profile. This approach provides more precise insights into the practical potential and limitations of using chitosan in industrial bread production.

## 2 Materials and methods

### 2.1 Materials

Wheat flour (Casillo), barley malt (Diamalteria), dextrose (Cargil), salt (Alos), brewer's yeast (Lesaffre), olive oil (Speroni), bakery adjuvant containing enzymes (Puratos) and water.

### 2.2 Manufacturing of soft bread samples

The soft bread samples were produced on an industrial scale by the food company Orva Spa, based in Bagnacavallo (RA, Italy). Dough was prepared using type 0 wheat flour (1880 g/L), barley malt (6 g/L), dextrose (8 g/L), salt (23 g/L), brewer's yeast (30 g/L), olive oil (57 g/L), bakery adjuvant containing enzymes (25 g/L) and water (1 L). The chitosan-enriched doughs were obtained by adding 0.75% and 1.5% of chitosan into the main ingredient mixture. The percentages of chitosan used in the breads were determined based on prior internal trials conducted by the company, which indicated that higher percentages resulted in excessively sticky dough and breads with unusual flavours. Chitosan (molecular weight: 100 KDa, deacetylated grade ≥90.0%) was purchased from Galeno SRL. The chitosan powder was added to the other ingredients using the company's own equipment. The ingredients were then mixed in a planetary mixer for about 10 min at two different speeds, the first lower (speed 2: 98 rpm/min, for 3 min) to allow the water to be absorbed by the ingredients and the second higher (speed 4: 145 rpm/min, for 7 min) to give the dough the right consistency by forming the gluten network. After the kneading phase, the doughs obtained were cut into 450 g balls and left to rise in moulds (24 × 11 cm) in a proofing cell at a temperature of 32 °C and relative humidity of 90% for 90 min. They were then baked in an industrial oven at a temperature of 210 °C for 31 min, with steam being blown in for the last 10 min. After baking, the soft bread samples were removed from the moulds and allowed to cool in trays at room temperature. After cooling, they were sliced and packed with ethanol (over at a concentration of no more than 2% of the product weight), under non-modified atmosphere using a film of COEX PP15 + LDPE 30 and stored for 60 days at room temperature (20 ± 5 °C).

For the dough, samples were collected before and after proofing and used to assess rheological properties.

For the bread, samples were collected during the shelf life (day 1, 20, 40 and 60) and used to assess texture and color characteristics, microbiological quality, prebiotic capacity, and volatile molecule profile.

### 2.3 Rheological measurements of bread dough

#### 2.3.1 Small deformation

The rheological measurements of the small deformations of the doughs before and after raising were carried out using a MCR 102 rotational rheometer (Anton Paar, Austria) with a parallel-plate geometry. The upper profiled 25 mm plate was lowered until the thickness of the sample was 2 mm and the excess was cut off. The exposed dough surfaces around gap edge were covered with a thin layer of mineral oil to prevent moisture loss during the test.

To identify the linear viscoelastic region in which the moduli are independent of strain, a strain sweep test was performed with a constant frequency of 10 Hz at 20 °C and a strain between 0.001 and 100 s^−1^ (Glicerina et al., 2018). Based on these results, a target strain of 0.01 % (within the linear range) was selected for the frequency sweep tests in a frequency range between 0.1 and 10 Hz (at 20 °C). The storage modulus G' (Pa), the loss modulus G'' (Pa), the complex shear modulus G∗ (Pa) and the phase shift angle δ (°) were determined using the rheometer software (RheoCompass, v. 1.14, Anton Paar, Austria) and compared at 1 Hz.

The results are the average of five replicates for each dough sample before and after the raising rest.

#### 2.3.2 Large deformation

The empirical rheological measurements of the large deformations in doughs on a laboratory scale were carried out with a TA.HDi 500 Texture Analyser (Stable Micro Systems, United Kingdom) after the raising rest. The Kieffer dough and gluten extensibility rig was used to measure the extensibility of the dough samples. Before the measurement, the dough was left to rest in the Teflon mold at 20 °C for 20 min. The test was performed under the following conditions: 25 kg load cell, 5 mm and 5 g probe calibration, 3.3 mm/s test speed, 10 mm/s post-test speed, 100 mm distance and an auto 5 g trigger force (Glicerina et al., 2018). The extension resistance (force in g) was determined by the maximum tensile strength and the extensibility (distance in mm) by the maximum distance before breakage. Texture Expert Exceed software (v. 2.64, Stable Micro Systems, United Kingdom) was used for data recording and processing.

The results are the average of five replicates for each dough sample.

### 2.4 Chemico-physical analysis of soft bread samples

#### 2.4.1 pH and water activity (a_w_)

The pH and a_w_ values of the soft bread samples were measured with a pH-meter (micropH2000, Crison, Barcelona, Spain) and an AquaLab device (Decagon, Pullman, WA). Every measurement was made in triplicate on days 1, 20, 40 and 60 of storage.

#### 2.4.2 Texture

The rheological properties of soft bread slice samples during storage were analysed using a Texture Analyser mod. TA-HDi 500 (Stable Micro System, United Kingdom) equipped with a 25 kg load cell and a 5 × 5 cm square probe. The texture profile analysis (TPA) was performed using a double compression cycle with a penetration depth of 40%, a test speed of 3 mm/s and a pause of 5 s between the two compressions (Balestra et al., 2011). The texture parameters considered were hardness (g) and springiness (adimensional). Hardness was determined by the maximum force measured during the first compression, while springiness was determined by the ratio of the distance between the maximum forces during the second and first compressions (Amani et al., 2022). Texture Expert Exceed software (v. 2.64, Stable Micro Systems, United Kingdom) was used to record and process the data.

Each sample was taken from the center part of the soft-sliced bread. The results are reported as the average of eight replicates for each sample and storage time.

#### 2.4.3 Height, porosity and color

The height of the bread slices and the porosity of soft bread crumb samples during storage was analysed using a computer vision system (CVS). Bread slices were placed in a dark room against a black background under controlled lighting conditions with four 6500 K fluorescent lamps (TL-D Deluxe, Natural Daylight D65, 18W/965, Philips, USA) placed 35 cm above the sample and inclined at an angle of 45°. The RGB images of the samples were acquired using a digital camera mod. D7000 (Nikon, Shinjuku, Japan) equipped with a 105 mm lens (mod. AF -S Micro Nikkor, Nikon, Japan). The following camera settings were used: manual mode, aperture f/20 and speed 1/2 s, ISO 100, no zoom, no flash, image resolution set to 300 dpi and saved in JPEG format. The camera was connected to a computer running Camera Control Pro software (version 2.7.0) (Nikon, Japan) to capture and view the digitised images.

**Fig. 1:**
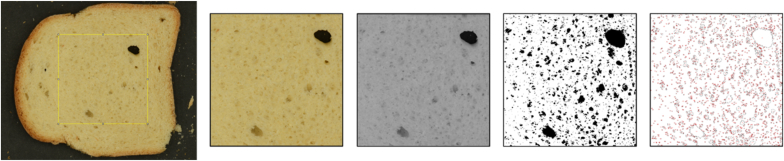
Example of image processing and analysis (from left): selection of area of interest (AOI); AOI cropping; conversion into greyscale image (8 bit); conversion to binary image; pores counting and analysis.

The acquired images were processed and analysed using ImageJ software (v. 1.49, NIH, USA). To determine the height (cm) of the bread slice samples, the scale was set using a graded scale ruler. The results are the average values of five replicates for each sample and storage time. The porosity of the bread crumb was determined according to the steps shown in Fig. 1. Briefly, the RGB images were cropped to the area of interest (AOI) corresponding to the central part of the bread slice (1800 x 1800 pixels). The images were then converted to an 8-bit greyscale image and then to a binary image and thresholded to measure the number and area of pores.

The samples were taken from the centre part of the soft-sliced bread. The results are the average values of three replicates for each sample and storage time.

The colour of bread crumb and crust was measured with a CR-400 colorimeter (Konica Minolta, Italy) equipped with a measuring port size of 50 mm of diameter. Colour was measured using the CIEL∗a∗b∗ scale. Colour change (ΔE) between the control bread (L∗_c_, a∗_c_, b∗_c_) sample and the chitosan-enriched samples were calculated using the equation (Silva et al., 2020):

$$\Delta E=\sqrt{{\left(L_{c}^{*}\;–\;L^{*}\right)}^{ˆ}2+{\left(a_{c}^{*}\;–\;a^{*}\right)}^{ˆ}2+{\left(b_{c}^{*}\;–\;b^{*}\right)}^{ˆ}2}$$

Numerical values of a∗ and b∗ were converted into hue angle (h°) and chroma (C∗) that represent the hue and the saturation index using the equations (McGuire, 1992):

$$h\;{}^{\circ}=\left(\tan^{-1}\left(b^{*}/a^{*}\right)/2\pi \right)\times 360$$

$${C\;}^{*}=\sqrt{{\left(a^{*}\right)}^{ˆ}2+{\left(b^{*}\right)}^{ˆ}2}$$

The measurement was carried out in triplicate on the crumb of two central slices of soft bread for each sample and storage time.

### 2.5 Microbiological analysis

To determine the microbial cell load of the soft bread samples, aerobic mesophilic bacteria and fungi (yeasts and moulds) were examined. Ten grams of each sample were homogenised in 90 mL of sterile 0.9% (p/v) NaCl solution using a stomacher for 2 min. Decimal dilutions were made into sterile 0.9% (p/v) NaCl solution and 0.1 mL aliquots were plated on Plate Count Agar (PCA), Yeast Extract, Peptone, Dextrose Agar (YPD) and Potato Dextrose Agar (PDA) (Oxoid) for the enumeration of mesophilic aerobic viable bacteria, yeasts and fungi, respectively. The PCA and YPD plates were incubated at 30 °C for 24–48 h, while the PDA plates were incubated at room temperature (25 °C) for 3–5 days. Colonies were then counted and expressed as colony forming units per gram (CFU/g) of samples. All counts were performed in triplicate on days 1, 20, 40 and 60 of storage.

### 2.6 Survival of probiotic bacteria in simulated intestinal fluid (SIF)

The ability of soft bread samples to promote the viability or growth of probiotic strains was investigated in simulated intestinal fluid (SIF: 0.1% w/v pancreatin, 0.15% w/v Oxgall bile salt, 100 mM phosphate salt buffer pH: 8) by plate counting according to Braschi et al. (2024) with modifications. The probiotic strains tested were *Bifidobacterium animalis* subsp. *lactis* BB-12 and *Lacticaseibacillus rhamnosus* GG. SIF enriched with 50 mg/mL soft bread samples was inoculated with 5–6 log CFU/mL of selected commercial probiotic strains. The inoculants were prepared by centrifugation (10000 g; 2 min; 4 °C) of overnight anaerobically grown cells of selected commercial probiotic strains and resuspended 1:1 in 0.9% (p/v) NaCl solution. After inoculation, SIF survival was monitored in response to supplementation with the different experimental conditions at 6 and 24 h by plate counting at MRS with 0.05% (p/v) cysteine HCl. The plates were incubated anaerobically at 37 °C for at least 24 h. Analyses were performed on soft bread samples on the 20th day of storage.

### 2.7 Volatile organic compounds (VOCs)

Volatile organic compounds (VOCs) compositions of different soft bread samples were qualitatively and quantitatively evaluated using head space solid phase microextraction using a gas chromatograph combined with a mass spectrometer detector (GCMS-SPME). A Carboxen®/DVB/PDMS, 50/30 μm fiber (SUPELCO) was used to perform the solid phase microextraction (SPME). The samples (3 g) were placed in vials and incubated for 10 min at 45 °C. Then the fiber was exposed to the vial headspace for 30 min at 45 °C. The volatile molecules adsorbed were transferred in the gas chromatograph (GC) injector port in splitless mode at 250 °C for 10 min. The headspace of the volatile compounds was analysed using Gas-Chromatography (GC) 6890N, Network GC System with mass spectrometry (MS) 5970 MSD (Agilent technologies). The column used was J&W CP-Wax 52 (50m × 320 μm × 1.2 μm) (Agilent technologies). The initial temperature was 50 °C for 1 min and then increased by 4.5 °C/min up to 65 °C. After that, the temperature increased by 10 °C/min up to 230 °C and remain at this temperature for 25 min. Gas-carrier was helium at 1.0 mL/min flow. Compounds were identified by comparison based on NIST 11 (National Institute of Standards and Technology) database. Analyses were performed in triplicate on days 1 and 60 of storage. The quantitative analysis were performed with the internal standard method, using 4-methyl-2-pentanol (6 mg/L), and expressed as equivalent ppm (ppm eq.).

### 2.8 Statistical analysis

Statistical analysis of the data was performed using Statistica 6.0 software (Statsoft Inc., USA). Results were expressed as means ± standard deviation. For the analysis of variance one-way (ANOVA), a significance level p ≤ 0.05 was set. In case of significant differences between samples, Tukey's post-hoc comparison test was used. The volatile molecule profiles were analysed using a principal component analysis (PCA) performed by Statistica software.

## 3 Results and discussion

### 3.1 Effect of chitosan on the rheological properties of soft bread dough samples

An accurate understanding of changes in dough rheological behaviour caused by the addition of chitosan in formulation is necessary to assess the processability of the product in the main industrial processes (Balestra et al., 2011).The results of the frequency sweep test conducted before and after raising phase on dough samples with (0.75% and 1.5%) and without chitosan (control) in formulation, are reported in Table 1.

**Table 1:** Influence of chitosan on dough samples fundamental rheological properties (mean values at 1 Hz of frequency).

Sample	G’ (Pa)	G’’ (Pa)	G∗ (Pa)	δ (°)
*Before raising*				
**Control**	10410.3 ± 1411.7 ^c, A^	4459.0 ± 339.1 ^c, A^	11327.6 ± 1427.9 ^c, A^	23.3 ± 1.3 ^a, B^
**0.75%**	21394.4 ± 1812.2 ^b, A^	8073.8 ± 348.5 ^b, A^	22869.0 ± 1816.9 ^b, A^	20.7 ± 0.8 ^b, B^
**1.5%**	26597.6 ± 2254.8 ^a, A^	9784.7 ± 556.7 ^a, A^	28344.0 ± 2270.9 ^a, A^	20.2 ± 1.0 ^b, A^
*After raising*				
**Control**	6322.1 ± 531.6 ^c, B^	3622.1 ± 342.6 ^c, B^	7286.5 ± 627.8 ^c, B^	29.8 ± 0.6 ^a, A^
**0.75%**	15388.6 ± 1114.5 ^b, B^	6425.9 ± 348.9 ^b, B^	16677.4 ± 1149.9 ^b, B^	22.7 ± 0.7 ^b, A^
**1.5%**	23518.8 ± 1503.8 ^a, B^	8889.5 ± 598.4 ^a, B^	25142.8 ± 1614.2 ^a, B^	20.7 ± 0.3 ^c, A^

Different lower case letters indicate significant differences between samples within the same raising rest. Different upper case letters indicate significant differences between the same sample before and after the raising rest.

As expected, the elastic modulus (G′) was higher than the viscous modulus (G″) in all dough samples and δ (phase shift angle) was between 0 and 45°, indicating a soft solid nature and visco-elastic behaviour due to the presence of the gluten network in the matrix (Ghoshal et al., 2017; Ma et al., 2021).

Before and after the raising rest, the addition of chitosan to the dough led to higher values of the modulus G′, G″ and G∗ (complex shear modulus) and a lower δ value than in the corresponding control samples. In general, a simultaneous increase in the modulus G∗ and a decrease in δ indicate an increasing degree of elasticity of the dough; all these results are associated with higher viscoelasticity and mechanical strength (Balestra et al., 2011; Ghoshal et al., 2017; Millar et al., 2019). This can make chitosan-enriched doughs difficult to mold, which may pose challenges in industrial processing (Balestra et al., 2011). These results are in line with previous studies by Ghoshal and Mehta (2019) and Zhu et al. (2024), who investigated chitosan-enriched cereal-based doughs. Chitosan was shown to have a swelling effect by filling the gluten network structure during dough formation and increasing the water holding capacity of the dough. A possible increase in the water holding capacity of the dough may have contributed to the increase in its viscoelasticity (Sansano et al., 2018; Zhu et al., 2024).

After 30 min of raising rest, a decrease in G′, G″ and G∗ with a simultaneous increase in δ was observed in almost all samples compared to the corresponding unleavened dough (before raising), indicating an improvement in industrial processing. This phenomenon could be due to the disruption of the increasingly cross-linked protein network by the formation of gas bubbles during dough fermentation, resulting in a weakened rheological structure (Ma et al., 2021). After the raising rest, there was a slight increase in δ in all samples, indicating a shift towards a more fluid system. However, this increase was less pronounced in the dough formulated with chitosan in relation to the added percentages, indicating a different degree of raising.

The results of the large deformation test of the dough samples agree with those of the fundamental rheological measurements. The addition of chitosan led to a remarkable increase in elongation resistance. Compared to the control (22.55 ± 3.68 g), the sample with 0.75% chitosan showed an extension resistance value of 27.10 ± 2.90 g, while the sample with 1.5% chitosan showed a significantly higher value of 41.70 ± 3.74 g. In addition, there were notable differences in extensibility, which also increased significantly in the enriched dough samples from 13.60 ± 0.99 mm (control sample) to 19.21 ± 9.71 mm and to 19.47 ± 4.60 mm for the samples with 0.75% and 1.5% chitosan, respectively. These results indicate that the chitosan makes the bread dough more compact and elastic. This result is similar to that of Ghoshal and Mehta (2019), who found an increase in extensibility values in chitosan-enriched dough samples compared to the control one. However, in their case, the dough with 1 % chitosan was the strongest and the most elastic compared to the doughs enriched with 0.5, 1.5 and 2%.

### 3.2 Effect of chitosan on the main quality properties of soft bread

#### 3.2.1 pH and water activity

Table 2 reports the data of the pH and a_w_ values measured on the bread samples under different experimental conditions. The pH values for the same sample showed no significant variations throughout the shelf life, except for the last time point (T60) for all samples and at T40 and T60 for the sample with 1.5% chitosan. However, pH variations were observed between the different types of soft breads. In fact, a higher chitosan content determined a significantly higher pH (p < 0.05). The cationic nature of chitosan could promote this effect, as when dissolved, it retains a hydrogen proton in its amine group, which can influence the final pH of the matrix. Indeed, Silva et al. (2020) also observed higher pH values in bread formulated with 1 and 2% chitosan.

**Table 2:** pH and water activity of the different bread samples during 60 days of storage.

	Time of storage (days)				
	Sample	T1	T20	T40	T60
**pH**	Control	5.55 ^c, A^ ± 0.04	5.55 ^c, A^ ± 0.05	5.57 ^c, A^ ± 0.03	5.67 ^c, B^ ± 0.02
	0.75%	6.02 ^b, A^ ± 0.03	6.02 ^b, A^ ± 0.01	6.04 ^b, A^ ± 0.03	6.20 ^b, B^ ± 0.03
	1.5%	6.23 ^a, A^ ± 0.01	6.24 ^a, A^ ± 0.05	6.29 ^a, A^ ± 0.02	6.42 ^b, B^ ± 0.05
**Water activity (a**_**w**_**)**	Control	0.932 ^b, A^± 0.008	0.931 ^b, A^ ± 0.003	0.930 ^b, A^ ± 0.002	0.934 ^b, A^ ± 0.002
	0.75%	0.929 ^b, A^ ± 0.001	0.936 ^b, A^ ± 0.002	0.933 ^b, A^ ± 0.002	0.939 ^b, A^ ± 0.002
	1.5%	0.949 ^a, A^ ±0.002	0.951 ^a, A^ ±0.001	0.955 ^a, A^ ± 0.002	0.955 ^a, A^ ± 0.002

Different lower case letters indicate significant differences between different samples within the same storage time. Different upper case letters indicate significant differences between the same sample over different storage days.

The a_w_ of the bread samples measured during the shelf life did not vary significantly over time with mean values of 0.931 ± 0.004, 0.934 ± 0.004 and 0.953 ± 0.003 for the control, 0.75% and 1.5% sample, respectively. The soft bread with 1.5% chitosan showed a significant higher a_w_ values compared to the other two samples. Ghoshal et al., 2017 suggested that chitosan acts as a molecular sponge, preventing the migration and redistribution of moisture between gluten and starch, and allowing the accumulation of moisture in interfacial regions where water molecules are more mobile. All these aspects play an important role on the final technological and microbiological characteristics of the bread.

#### 3.2.2 Texture, slice height, pores features and color of bread samples

In general, the crumb of a soft bread type can be described as tender, moist and airy. It typically has a homogeneous, fine texture with small, evenly distributed air pores that give it a slightly elastic and soft consistency (Petrusha et al., 2018). Table 3 shows the hardness and springiness values of soft bread slice samples, with and without chitosan, during storage. Hardness is defined as the force required to compress the food with the cheek teeth during the first bite, while springiness refers to the elastic recovery of the bread between the first and second compression (Silva et al., 2020). The control bread had the lowest hardness and the highest springiness values during the entire storage period. On the other hand, the addition of chitosan in the recipe contributed to increase the hardness and to reduce the springiness of the bread samples, especially of those containing 1.5% chitosan. Thus, chitosan seems to influence the texture of the bread samples in a concentration-dependent manner. Similar results were also reported in other studies in which the use of chitosan increased the firmness of the bread proportionally to its added amount (Ghoshal and Mehta, 2019; Lafarga et al., 2013; Silva et al., 2020; Zhu et al., 2024). Due to its hydrophilic properties, chitosan, when added to a bread recipe, can bind water molecules as a fibre, facilitating the dehydration both from starch and gluten and moisture migration from crumb to crust resulting in a firmer texture (Ghoshal and Mehta, 2019). In addition, the increase in overall firmness with increasing chitosan content could also be due to a lower specific volume development as showed by the significantly lesser slice height (Table 3) that could be attributed to chitosan's ability to interact with lipids and prevent amylose–lipid complexation, thereby facilitating the rate of firming. Alternatively, chitosan could have interfered with microbial fermentation (Ghoshal and Mehta, 2019; Silva et al., 2020) which can determine a diminished dough development and limited expansion of the final product (Lafarga et al., 2013). As expected, all samples showed a time-dependent increase in hardness and a decrease in springiness during storage, which is probably due to the bread staling process that occur during shelf-life (Ghoshal et al., 2016). The bread sample with 1.5% chitosan showed the highest increase in firmness from T1 to T60 (+103.36%) compared to the other samples (control: +74.97%; 0.75%-chitosan bread: +63.82%). The size of the pores of the bread crumb significantly influences both the texture and the mouthfeel (Balestra et al., 2011). The results in Table 3 show that, across all time intervals, the addition of chitosan to the bread formulations at concentrations of 0.75% and 1.5% resulted in a higher number of pores and a larger average pore area compared to the control sample. Therefore, the control sample exhibited finer and compact alveolation throughout the storage period compared to the chitosan-enriched ones. These results could be attributed to the water-binding and CO_2_-retaining properties of chitosan when included in bread formulation (Hastarini and Ayudiarti, 2019), which promoted the development of larger pores during the rising and baking of the product. No significant differences were observed in pore area over the storage time, although some samples showed slight variations in the number of pores, which could be associated with bread staling.

**Table 3:** Characteristics of texture (hardness and springiness), slice height, porosity (number and area of pores), crumb and crust colour (ΔE, hue angle and chroma) of control, 0.75% and 1.5% chitosan bread samples during storage.

Sample	T1	T20	T40	T60
*Hardness (g)*				
**Control**	61.32 ± 3.09 ^c, D^	92.27 ± 12.04 ^b, C^	105.11 ± 9.58 ^b, B^	107.29 ± 3.07 ^c, A^
**0.75%**	74.15 ± 3.44 ^b, D^	95.60 ± 9.06 ^b, C^	110.49 ± 4.14 ^b, B^	121.47 ± 10.14 ^b, A^
**1.5%**	80.40 ± 8.84 ^a, C^	106.92 ± 13.28 ^a, B^	160.21 ± 12.77 ^a, A^	163.50 ± 7.07 ^a, A^
*Springiness (adimensional)*				
**Control**	0.94 ± 0.01 ^a, A^	0.91 ± 0.02 ^a, B^	0.92 ± 0.03 ^a, B^	0.90 ± 0.01 ^a, C^
**0.75%**	0.91 ± 0.01 ^b, A^	0.86 ± 0.03 ^b, B^	0.87 ± 0.01 ^b, B^	0.87 ± 0.04 ^b, B^
**1.5%**	0.89 ± 0.01 ^c, A^	0.86 ± 0.01 ^b, B^	0.87 ± 0.03 ^b, B^	0.87 ± 0.01 ^b, B^
*Height (cm)*				
**Control**	10.72 ± 0.20 ^a, A^	9.85 ± 0.54 ^a, B^	9.21 ± 0.18 ^a, C^	9.24 ± 0.22 ^a, C^
**0.75%**	9.66 ± 0.51 ^b, A^	9.36 ± 0.32 ^b, A^	8.97 ± 0.29 ^b, B^	8.86 ± 0.36 ^b, B^
**1.5%**	9.41 ± 0.29 ^c, A^	8.45 ± 0.48 ^c, B^	7.96 ± 0.22 ^c, C^	7.70 ± 0.23 ^c, C^
*Number of pores*				
**Control**	1554.00 ± 131.52 ^c, B^	1526.50 ± 108.55 ^c, B^	1570.33 ± 58.59 ^c, B^	2071.50 ± 119.09 ^b, A^
**0.75%**	2462.33 ± 199.61 ^b, A^	2493.33 ± 265.21 ^b, A^	2315.50 ± 121.92 ^b, A^	2413.00 ± 291.60 ^a, A^
**1.5%**	2947.33 ± 152.00 ^a, A^	3035.00 ± 221.00 ^a, A^	2642.67 ± 210.04 ^a, B^	2578.00 ± 130.22 ^a, B^
*Area of pores (pixel)*				
**Control**	192.93 ± 30.53 ^c, A^	192.39 ± 18.34 ^b, A^	184.91 ± 32.27 ^c, A^	195.04 ± 10.83 ^c, A^
**0.75%**	216.90 ± 16.38 ^b, A^	203.10 ± 9.39 ^b, A^	200.32 ± 14.68 ^b, A^	212.52 ± 14.24 ^bc, A^
**1.5%**	249.36 ± 13.33 ^a, A^	245.39 ± 26.49 ^a, A^	239.04 ± 25.68 ^a, A^	221.85 ± 17.59 ^ab, A^
*ΔE crumb*				
**Control**	–	–	–	–
**0.75%**	3.71 ± 0.37 ^b, A^	3.94 ± 0.90 ^b, A^	4.27 ± 1.25 ^a, A^	4.61 ± 0.52 ^b, A^
**1.5%**	4.39 ± 0.83 ^a, C^	5.82 ± 0.64 ^a, B^	5.10 ± 0.61 ^a, B^	8.94 ± 0.65 ^a, A^
*ΔE crust*				
**Control**	–	–	–	–
**0.75%**	2.70 ± 1.28 ^b, A^	3.86 ± 1.35 ^b, A^	3.10 ± 1.29 ^b, A^	2.56 ± 1.19 ^b, A^
**1.5%**	4.33 ± 0.63 ^a, B^	6.07 ± 3.16 ^a, A^	5.28 ± 1.02 ^a, A^	5.62 ± 1.53 ^a, A^
*h° (hue angle) crumb*				
**Control**	96.54 ± 0.51 ^a, A^	96.22 ± 0.34 ^a, AB^	95.55 ± 0.52 ^a, BC^	95.06 ± 0.46 ^a, C^
**0.75%**	93.69 ± 0.29 ^b, A^	92.81 ± 0.81 ^b, AB^	91.69 ± 0.71 ^b, C^	92.14 ± 0.40 ^b, BC^
**1.5%**	93.31 ± 0.57 ^b, A^	92.02 ± 0.99 ^b, B^	91.52 ± 0.40 ^b, B^	86.95 ± 0.31 ^c, C^
*h° (hue angle) crust*				
**Control**	69.53 ± 0.77 ^a, B^	70.19 ± 3.02 ^a, AB^	72.36 ± 3.48 ^a, AB^	73.74 ± 1.95 ^a, A^
**0.75%**	70.71 ± 1.12 ^a, AB^	69.25 ± 1.55 ^a, B^	72.10 ± 0.57 ^a, A^	71.81 ± 0.40 ^b, A^
**1.5%**	70.65 ± 0.52 ^a, A^	70.55 ± 2.27 ^a, A^	72.56 ± 1.73 ^a, A^	69.76 ± 0.38 ^c, A^
*C∗ (chroma) crumb*				
**Control**	14.33 ± 0.76 ^b, B^	14.72 ± 0.34 ^b, AB^	15.62 ± 0.50 ^b, A^	15.38 ± 0.74 ^c, A^
**0.75%**	17.71 ± 0.40 ^a, C^	18.25 ± 0.88 ^a, B^	19.89 ± 1.00 ^a, A^	19.04 ± 0.42 ^b, A^
**1.5%**	17.35 ± 0.79 ^a, C^	18.66 ± 1.20 ^a, BC^	19.92 ± 0.70 ^a, B^	21.81 ± 0.53 ^a, A^
*C∗ (chroma) crust*				
**Control**	34.48 ± 0.39 ^a, A^	34.47 ± 1.00 ^a, A^	31.91 ± 1.24 ^a, A^	32.42 ± 3.97 ^a, A^
**0.75%**	33.19 ± 1.34 ^b, A^	32.57 ± 0.68 ^b, AB^	32.41 ± 0.48 ^a, AB^	31.34 ± 1.14 ^a, B^
**1.5%**	31.06 ± 0.30 ^c, AB^	31.99 ± 0.16 ^b, A^	31.46 ± 1.60 ^a, AB^	30.30 ± 0.61 ^a, B^

Different lower case letters indicate significant differences between different samples within the same storage time. Different upper case letters indicate significant differences between the same sample over different storage days.

**Fig. 2:**
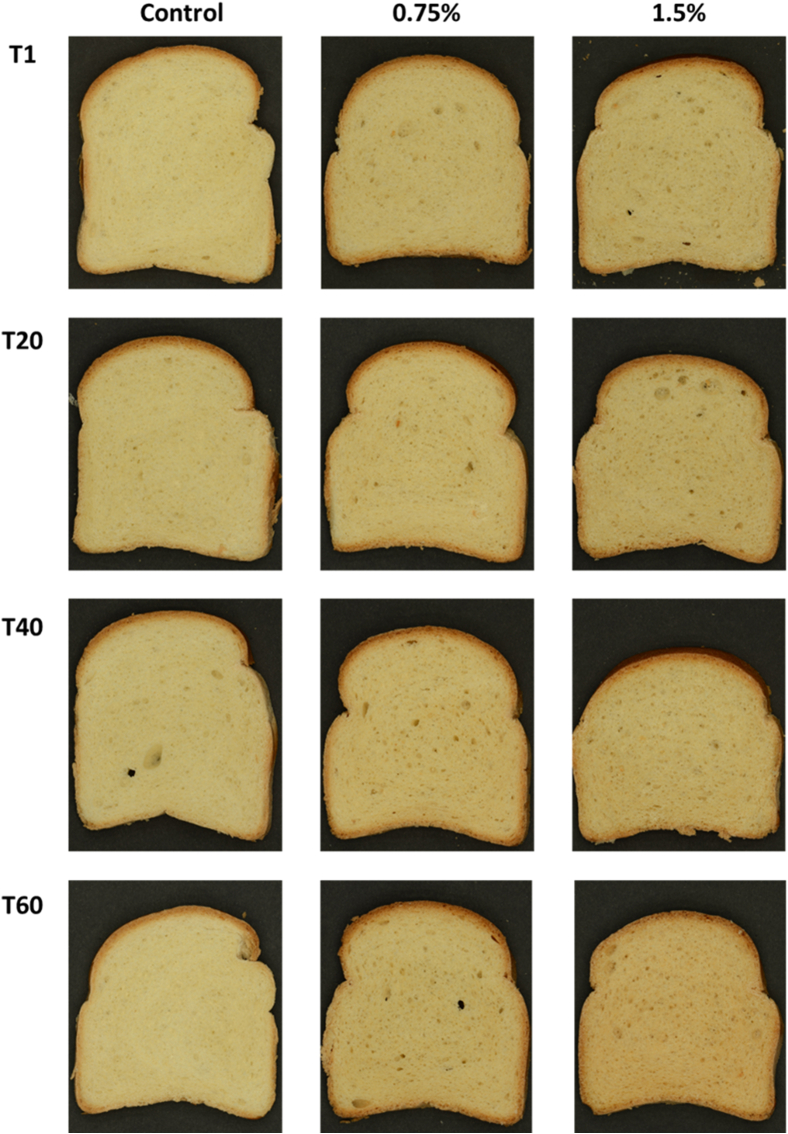
Visual comparison of bread slice samples formulated without chitosan (control) and with 0.75% and 1.5% chitosan concentrations during storage.

An increase in the percentage of chitosan also led to a more pronounced coloration of the bread crumb, as observed visually (Fig. 2) and measured by colorimetric analysis (Table 3). The ΔE results for both crumb and crust, which is attributed to colour variation, showed some differences between the control sample and the chitosan-enriched ones that were proportional to the amount of chitosan added. The crumb of bread containing chitosan presented a reduced hue angle (h°), indicating a shift towards orange/red tones compared to the control sample, which was more yellowish. A slight decrease in yellow colour towards more orange/red tones was observed in the crumb in all samples during the shelf life. Regarding the bread crust, the h° value slightly decreased with increasing chitosan concentration from 0.75% to 1.5%, but this change was only observed after a 60-day storage period. The increase in chroma (C∗) values with increasing chitosan concentration directly indicates an increase in colour saturation. Higher chroma values reflect colours of greater purity and intensity and are consistent with the other chromatic parameters, according to which higher concentrations of chitosan resulted in a more saturated colour appearance of the bread crumb. In general, the crusts of the samples were more colour saturated than those of the crumbs. In contrast to the crumb, the C∗ value of the crust tended to decrease with increasing chitosan concentration. Previous studies have shown that the addition of chitosan to wheat flour bread recipes led to a darker colour of both crust and crumb, which is attributed to an increased intensity of Maillard reactions during baking (Kerch et al., 2008, 2010). In fact, glucosamine, the building block of chitosan, is a significantly unstable molecule that can produce a high level of Maillard and caramelization products already at 37 °C (Hrynets et al., 2015). Since soft bread based on refined wheat flour (control sample) is typically characterised by a white crumb and a light-brown crust, the colour changes caused by the addition of chitosan may have an impact on sensory appeal, especially at higher concentrations such as 1.5%. However, these potential sensory side effects should be evaluated through a consumer panel test.

### 3.3 Microbiological analyses

Microbial spoilage, especially fungal spoilage, is the main cause of the significant wastes in packaged bakery products. Therefore, each sample was analysed from a microbiological point of view to evaluate the load of moulds, yeast, and mesophilic bacteria during the entire shelf life (T1, T20, T40 and T60). As shown in Table 4, moulds (log CFU/g) were below the limit of quantification at all the storage times in all the sample considered. In terms of yeast concentration, while it remained below the limit of quantification throughout the entire shelf life in control samples, breads made with chitosan, particularly those containing 1.5%, exhibited higher levels of yeasts. Specifically, the yeast content in samples with 0.75% chitosan reached 1.3 log CFU/g only at T60, whereas the sample with 1.5% chitosan started at that concentration at T1 and increased to 2.9 log CFU/g by T60. The total mesophilic microorganisms were below the limit of quantification in the control sample and in bread made with 0.75% chitosan at T1, while 1.2 log CFU/g were quantified in bread with 1.5% chitosan. During the shelf life, the total mesophilic content increased in all bread samples, ranging from 3.0 to 3.5 log CFU/g, with the highest levels consistently observed in bread containing 1.5% chitosan. Several studies highlighted how the addition of chitosan inhibits bacterial growth in food products, such as noodle dough, cheeses, salad dips and ethnic foods (Altieri et al., 2005; Guan and Feng, 2022; Muanprasat and Chatsudthipong, 2017; Olaimat et al., 2023; Zhang et al., 2020). The antimicrobial and antifungal activity of chitosan caused by cationic moiety is promoted in an acidic media. In fact, at pH values below the pKa of chitosan, protonated amino groups bind the negatively charged carboxyl groups of lipopolysaccharides and peptidoglycans of the bacterial cell wall, leading to permeabilization and destruction of external membranes (Zhou et al., 2021). At the same time, chitosan molecular weight and degree of acetylation have an impact on the final antimicrobial activity (Rakkhumkaew and Pengsuk, 2018).

**Table 4:** Microbial cell load (log CFU/g) of fungi (moulds and yeast) and aerobic mesophilic bacteria in soft bread samples during shelf life (T1, T20, T40 and T60).

Cell load viability (log CFU/g)				
**Moulds**	**T1**	**T20**	**T40**	**T60**

Control	*-*	*-*	*-*	*-*
0.75%	*-*	*-*	*-*	*-*
1.5%	*-*	*-*	*-*	*-*

**Yeasts**	**T1**	**T20**	**T40**	**T60**

Control	*-*	*-*	*-*	*-*
0.75%	*-*	*-*	*-*	1.3 ± 0.1 ^b^
1.5%	1.3 ± 0.2 ^C^	1.8 ± 0.2 ^B^	2.8 ± 0.2 ^A^	2.9 ± 0.2 ^a, A^

**Aerobic mesophilic bacteria**	**T1**	**T20**	**T40**	**T60**

Control	*-*	1.5 ± 0.4 ^b, C^	2.6 ± 0.4 ^b, B^	3.1 ± 0.1 ^b, A^
0.75%	*-*	1.1 ± 0.4 ^b, C^	2.1 ± 0.3 ^b, B^	3.0 ± 0.1 ^b, A^
1.5%	1.2 ± 0.6 ^C^	2.2 ± 0.3 ^a, B^	3.2 ± 0.3 ^a, A^	3.5 ± 0.2 ^a, A^

Different lower case letters indicate significant differences between different samples within the same storage time. Different upper case letters indicate significant differences between the same sample over different storage days.”–“: below the limit of detection (<1 Log CFU/g).

The data collected in this study suggested that chitosan (MW: 100 kDa; DA ≥ 90%) did not inhibit the growth of indigenous microorganisms. The first reason can be attributed to the higher pH of chitosan bread (above 6) that can have reduced chitosan antimicrobial properties. Second, carbohydrates, proteins, fats, fiber, minerals, vitamins, and salts, which are present in bread, can interfere with chitosan antibacterial activity. In fact, Devlieghere et al. (2004) and No et al. (2007), reported that starch, whey protein, and NaCl can negatively impact the antimicrobial activity of chitosan, while oil has no effect. At the same time, the higher counts of yeasts and aerobic mesophilic bacteria observed in 1.5% chitosan-bread may be the results of the higher a_w_ values (around 0.949 compared to 0.932 and 0.929 in control and 0.75% chitosan bread during the entire shelf life of the products) which can allow the growth of these type of microorganisms. Overall, it is important to mention that up to 60 days the microbial growth has not exceeded the acceptable limits considered for these type of products (below 4 and 3 log CFU/g for aerobic mesophilic bacteria and yeasts, respectively (CeIRSA, 2013).

### 3.4 Prebiotic activity

**Fig. 3:**
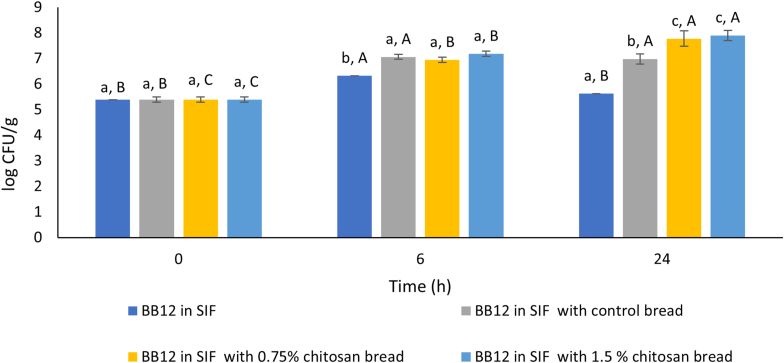
Survival (log CFU/g) of *Bifidobacterium animalis* subsp. *lactis* BB-12 in simulated intestinal fluid (SIF) with control bread, 0.75% chitosan bread and 1.5% chitosan bread. SIF without supplementation served as the control condition. Survival assessments were conducted on the 20th day of storage. Different lower case letters indicate significant differences between different samples within the same storage time. Different upper case letters indicate significant differences between the same sample over different storage days.

**Fig. 4:**
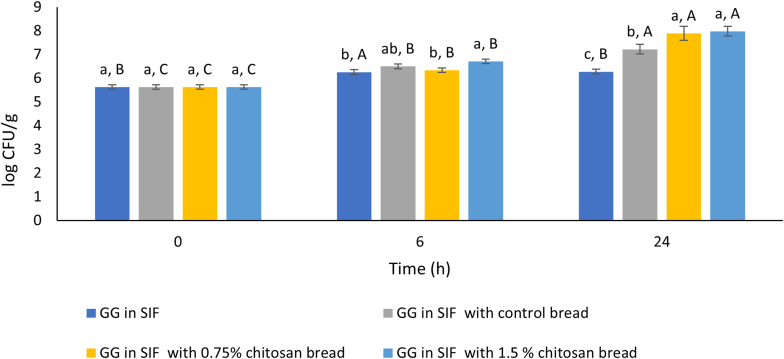
Survival (log CFU/g) of *Lacticaseibacillus rhamnosus* GG in simulated intestinal fluid (SIF) with control bread, 0.75% chitosan bread, 1.5% chitosan bread. SIF without supplementation served as the control condition. Survival assessments were conducted on the 20th day of storage. Different lower case letters indicate significant differences between different samples within the same storage time. Different upper case letters indicate significant differences between the same sample over different storage days.

In recent years, the prebiotic activity of chitosan has been widely demonstrated, indicating its promising use in the food sector (Das et al., 2022; Guan and Feng, 2022). Therefore, the bread samples were tested for their ability to promote the growth of selected probiotic strains (*Bifidobacterium animalis* subsp. *lactis* BB-12 and *Lacticaseibacillus rhamnosus* GG) in simulated intestinal fluid (SIF) during 0, 6 and 24 h of incubation. As shown in Fig. 3, an increase in the microbial load of *B. lactis* BB-12 was observed after 6 h of incubation in SIF in all bread samples, with and without chitosan, compared to the condition with SIF alone. This suggests that the increased viability of *B. lactis* BB-12 was likely due to the food matrix itself rather than the addition of chitosan. Conversely, after 24 h of incubation, bread samples with chitosan (regardless of the concentration used, 0.75 or 1.5%) showed higher counts of *B. lactis* BB-12 (7.8 and 7.9 Log CFU/g compared to the control, 7 Log CFU/g), suggesting enhanced probiotic growth and viability associated with the incorporated bioactive compound. Regarding the cell viability of *L. rhamnosus* GG (Fig. 4) no significant differences were observed between the different samples during the first 6 h of incubation in SIF, unlike *B. lactis* BB-12, even when considering the activity of *L. rhamnosus* GG in SIF without bread. However, also in this case, a significant increase (7.9 and 8.0 Log CFU/g) was observed after 24 h incubation in bread samples with chitosan (0.75 and 1.5%, respectively), compared to control one (7.2 Log CFU/g), indicating a significant prebiotic activity of the product. Even *in vivo* trial, an increase in the abundance of Lactobacilli upon treatment with chitosan was reported in weaned piglets by Hou et al. (2021). However, according to literature data, chitosan oligosaccharides (below 3.9 kDa) possess higher prebiotic activity than chitosan (Guan and Feng, 2022). Our study therefore represents a further step forward in the evaluation of the functionality of chitosan in bread, especially about its prebiotic potential, also with regard to the development of a functional food. Indeed, most studies on chitosan in bread mainly focus on analysing its technological properties and antimicrobial potential (Ghoshal and Mehta, 2019; Lafarga et al., 2013; Silva et al., 2020; Zhu et al., 2024). In this context, several studies highlight the prebiotic potential of chitosan, not so much when it is used as an ingredient, but rather when it is employed as an encapsulation material for probiotic microorganisms. In fact, it has been shown to promote the viability of probiotics even after simulated digestive processes (Adilah et al., 2022; Afzaal et al., 2022; Maleki et al., 2022).

### 3.5 Volatile organic compounds (VOCs) composition

**Fig. 5:**
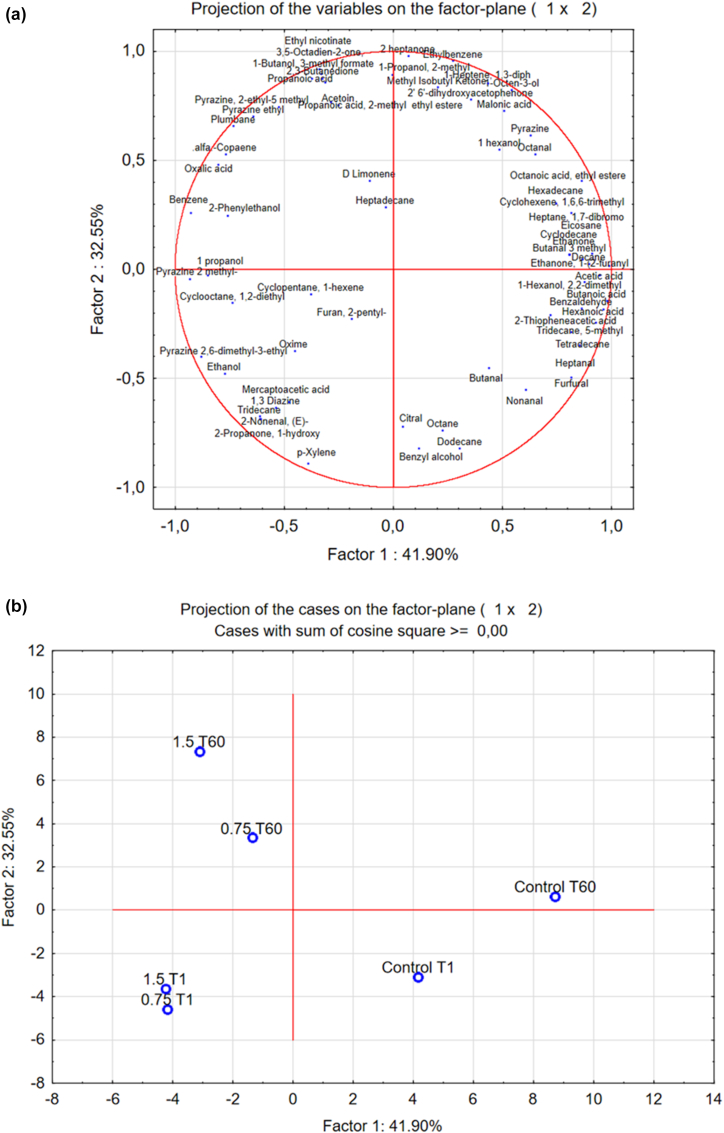
Plot of variables (a) and cases (b) obtained by PCA elaboration of the total volatile molecules characterizing the soft breads after 1 day and 60 days of storage. Control: control bread; 0.75: 0.75% chitosan bread and 1.5: 1.5% chitosan bread.

Among the various attributes of bread, odor quality holds significant importance as it is among the initial characteristics perceived by consumers. Solid phase microextraction (SPME) combined with GC-MS is usually selected as the technique to determine bread volatile compounds. As reported in Table 5, around 67 volatile compounds, including alcohols, aldehydes, ketones, acids, hydrocarbons, pyrazines, and furans were detected in all the samples, at the beginning (T1) and at the end of storage (T60). The most abundant class of compounds were alcohols (76.70–48.11 ppm eq.), hydrocarbons (14.95–9.84 ppm eq.), acids (6.07–3.56 ppm eq.), and aldehydes (4.12–2.00 ppm eq.). On T1, no significant differences were observed among samples for total hydrocarbons, while breads made with chitosan (regardless the concentration applied) presented higher alcohols (75.73–76.70 compared to 48.11 ppm eq.) and lower acids (3.56–3.74 compared to 5.58 ppm eq.) concentrations than control. On day 60, only alcohols were maintained higher in bread made with chitosan (54.66–59.04 compared to 49.67 ppm eq.) while higher hydrocarbons were observed in the control (14.95 compared to 9.84–10.36 ppm eq.). To better understand the effect related to both the addition of chitosan (0.75 or 1.5%) and the storage time on the aroma profiles of the different soft bread samples, a principal component analysis (PCA) was performed with the volatilome data measured after 1 and 60 days of storage (Fig. 5). From Fig. 5 it is possible to highlight differences between samples as a function of their different formulation (with or without chitosan). The compounds that mainly rappresented the cluster of chitosan-bread at day 1 were ethanol, (E)-2-nonenal, 2,6-dimethyl-3-ethylpyrazine and 2-pentylfuran. The presence of ethanol in all the three bread samples is associated to the addition of this preservative during the packaging process phase. However, even if added at the same concentration, it seems that bread made with chitosan could retain more ethanol which is then observed in higher amount on these samples headpsace. Although no data are reported in literature supporting this statement (or more in general no studies were performed on evaluating VOCs in bread made with chitosan), chitosan which contains amino groups could interact with hydroxyl groups through charge interactions (Chetouani et al., 2017) which can be easily broken during the analyses cunducted at 45 °C. A second hypothesis is that higher level of ethanol can derive from microbial fermentation which could have been more intense in chitosan samples. Alternatively, ethanol may have been generated during the storage due to the higher yeast count observed.

**Table 5:** Volatile organic compounds (VOCs) of control bread, 0.75% and 1.5% chitosan bread samples at the beginning (T1) and at the end of storage (T60). Data expressed in equivalent parts per million (ppm eq.) are the mean values of three replicates. Standard deviation observed ranged between 3 and 5%.

		Control T1	0.75% T1	1.5% T1	Control T60	0.75% T60	1.5% T60
Aldehydes	Benzaldehyde	1.40	0.88	0.73	1.22	0.94	0.82
	Butanal	0.23	0.03	0.03	0.03	–	–
	Butanal 3 methyl	–	–	–	0.03	–	–
	Heptanal	0.17	0.15	0.14	0.22	0.15	0.10
	Nonanal	0.78	0.74	0.83	0.95	0.56	0.61
	2-Nonenal, (E)-	–	0.05	0.04	–	–	–
	Octanal	0.06	0.03	0.04	0.06	0.05	0.06
	Furfural	1.48	0.68	0.37	1.25	0.30	0.13
Alcohols	1-Butanol, 3-methyl formate	1.15	1.22	1.32	1.29	1.50	1.53
	1-Hexanol	0.63	0.64	0.64	0.75	0.64	0.71
	1-Hexanol, 2,2-dimethyl	0.10	0.08	0.06	0.10	0.09	0.07
	1-Octen-3-ol	0.12	0.06	0.08	0.15	0.13	0.16
	1-Propanol	0.02	0.04	0.03	0.02	0.02	0.03
	1-Propanol, 2-methyl	0.13	–	–	0.11	0.14	0.43
	Benzyl alcohol	0.10	0.10	0.09	0.08	0.05	0.07
	2 Phenylethanol	1.28	1.75	2.47	1.26	1.52	2.50
	Ethanol	44.58	71.84	72.01	45.91	50.57	53.54
Ketones	2,3-Butanedione	–	–	–	–	0.04	0.14
	Methyl Isobutyl Ketone	0.04	0.04	0.04	0.06	0.06	0.06
	Acetoin	0.12	0.07	0.07	0.08	0.07	0.85
	2-Heptanone	–	–	–	0.05	0.06	0.10
	Ethanone	–	–	–	0.07	–	–
	Ethanone, 1-(2-furanyl	0.44	0.25	0.17	0.46	0.25	0.26
	3,5-Octadien-2-one,	–	–	–	–	0.03	0.08
Hydrocarbons	Decane	0.06	0.05	0.04	0.10	0.06	0.04
	Dodecane	2.36	2.79	2.79	2.92	1.88	1.55
	Tetradecane	5.15	4.10	4.53	5.77	3.44	4.16
	Hexadecane	1.20	0.99	1.02	1.58	0.90	1.32
	Ethylbenzene	0.06	0.02	0.02	0.11	0.12	0.15
	Benzene	–	0.06	0.08	–	0.06	0.08
	1-Heptene, 1,3-diph	0.11	0.09	0.09	0.19	0.18	0.18
	Cyclopentane, 1-hexene	0.03	0.20	0.02	0.03	0.20	0.02
	Cyclooctane, 1,2-diethyl	0.46	0.52	0.37	0.02	0.45	0.41
	Cyclodecane	–	–	–	0.49	–	–
	Octane	0.09	0.07	0.05	0.05	0.06	0.03
	p-Xylene	0.21	0.24	0.26	0.11	0.10	0.10
	2′ 6′-dihydroxyacetophenone	0.37	0.39	0.41	0.59	0.47	0.58
	Heptadecane	0.09	0.04	0.04	0.05	0.30	0.04
	Tridecane, 5-methyl	0.18	0.13	0.15	0.23	0.13	0.12
	2-Propanone, 1-hydroxy	–	0.07	0.07	–	–	–
	Eicosane	0.19	0.11	0.17	0.53	0.15	0.15
	Cyclohexene, 1,6,6-trimethyl	0.35	0.30	0.33	0.57	0.32	0.38
	Heptane, 1,7-dibromo	1.17	0.91	1.07	1.61	1.02	1.05
	Tridecane	–	0.02	0.03	–	–	–
Diazines	Pyrazine	0.32	0.03	0.03	0.33	0.37	0.27
	Pyrazine 2 methyl-	–	0.31	0.23	–	0.20	0.20
	1,3 Diazine	–	0.12	0.05	–	–	–
	Pyrazine, 2,6-dimethyl 3 ethyl	0.06	0.09	0.09	0.05	0.07	0.07
	Pyrazine ethyl	–	0.09	0.05	–	0.10	0.22
	Pyrazine, 2-ethyl-5 methyl	–	0.04	0.04	–	0.05	0.21
Terpenes	Citral	0.16	0.09	0.05	–	–	–
	D Limonene	0.05	0.02	0.04	0.03	0.30	0.05
	alfa-Copaene	–	0.04	0.09	–	0.11	0.11
Acids	Mercaptoacetic acid	0.14	0.17	0.19	0.13	0.11	0.15
	2-Thiopheneacetic acid	2.80	1.11	1.27	2.17	1.26	1.76
	Butanoic acid	0.15	0.00	–	0.21	–	–
	Hexanoic acid	0.15	0.00	–	0.18	–	–
	Propanoic acid	0.00	0.00	–	–	0.05	0.05
	Oxalic acid	0.00	0.77	1.21	–	0.76	1.83
	Octanoic acid, ethyl ester	0.15	0.11	0.11	0.18	0.13	0.15
	Acetic acid	1.59	0.98	0.93	1.77	1.22	1.06
	Malonic acid	0.60	0.42	0.03	0.87	0.60	0.95
	Propanoic acid, 2- ethyl ester	0.00	0.00	–	–	–	0.12
Other compounds	Ethyl nicotinate	0.00	0.00	–	–	0.12	0.17
	Furan, 2-pentyl	0.45	0.24	0.19	0.03	0.22	0.23
	Oxime	0.24	0.79	0.84	0.61	0.61	0.42
	Plumbane	0.00	0.79	0.91	0.16	1.10	1.83

- Not detected.

The presence of (E)-2-nonenal, 2-pentylfuran and 2,6-dimethyl-3-ethylpyrazine, are associated with odor notes of malt, green, tallow, sweet and potato, and are usually found in different types of bread. The first compound is generated by enzymatic oxidation of linoleic acid during the storage of flours and during dough mixing and fermentation, while 2-pentylfuran can be produced either by microbial fermentation or form the baking process (Cho and Peterson, 2010; De Luca et al., 2021). Pyrazines were also more abundant in chitosan samples and their concentration was still higher after 60 days of storage. Chitosan samples mantained also a higher level of ketones like 2,3-butanedione (diacetyl) and alcohols like 2-phenylethanol, both deriving from microbial fermentation. The first compound is responsible for imparting a buttery or creamy aroma and flavor to baked goods (Cho and Peterson, 2010) while 2-phenylethanol, already mentioned in bread samples by Annan et al. (2003) and De Luca et al. (2021) is responsible for rose/floral odors. Interestigly, pyrazines, aldheydes and ketones, such as diacetyl, were also reported by Hrynets et al. (2015) during incubation of glucosamine at 37 °C, therefore deriving from chemical rearrangments of the unstable building block of chitosan. On the other hand, control samples were characterized by a higher level of acetic acid (characteristic odor of acid, pungent, rancid and sweaty) and certain aldehydes (heptanal: green, rancid, grassy – nonanal: soapy, fruity, rose - benzaldehyde: almondy, sweet and cherry). Acetic acid is a microbial metabolite commonly found in sourdough which can confer antimicrobial properties improving the shelf life of the bread, while aldehydes are the result of lipid oxidation and/or fermentation (De Luca et al., 2021). Regarding acetic acid, the microbial counts of control samples do not suggest possible microbiological production after baking. It is more likely that acetic acid was produced during dough preparation. Given this, the higher ethanol content observed in chitosan bread and higher acetic acid in control sample may also be due to a different microbiota or metabolome imparted by the presence of chitosan.

## 4 Conclusions

The results observed in this work align well with existing literature data, particularly concerning the impact of chitosan on the technological properties of dough and bread. The addition of chitosan to soft bread formulation increases the viscoelasticity, firmness, and extensibility of the dough, making it more elastic but also more challenging to mold and process it industrially. Chitosan-enriched bread exhibits higher pH, water activity, firmness, and pore size, along with a reduced slice height and a more pronounced color shift due to increased Maillard reactions. From a microbiological point of view, the products obtained with chitosan were within the acceptability limits for this type of food. However, limited effects were observed on controlling the spoiling microbiota, which developed more rapidly in chitosan-enriched bread. This aspect is not in line with literature data where the phenomenon was partially described due to the use of model systems or challenge test with target microorganisms. In our case, the development of the microbiota already present in the industrial product was considered during the shelf life study that lasted 60 days. This aspect needs to be considered when chitosan is used (added) as ingredient in bread formulation, especially at industrial level. At the same time, the addition of chitosan (despite the concentration applied) allowed to obtain bread with higher prebiotic activity and that developed aroma compounds characteristic of soft wheat bread. This study, therefore, shows that although the addition of chitosan had significant technological limitations, both in terms of the workability of the dough and the texture of the baked bread, interestingly, the percentages used did not lead to an off-flavour in the final product. The use of chitosan could also functionalise the bread and give it increased prebiotic activity, which could be of great benefit to today's consumers. Overall, these data provide useful information on the actual industrial application of chitosan in bread formulation, which must be taken into account in the production of innovative products with satisfactory technological, microbiological and organoleptic properties.

## CRediT authorship contribution statement

**Margherita D'Alessandro:** Investigation, Data curation, Writing – original draft, Writing – review & editing, Visualization. **Maria Alessia Schouten:** Investigation, Data curation, Writing – original draft, Writing – review & editing, Visualization. **Davide Gottardi:** Conceptualization, Validation, Data curation, Writing – original draft, Writing – review & editing. **Sara Cortesi:** Conceptualization, Investigation, Resources, Writing – review & editing. **Santina Romani:** Methodology, Validation, Resources, Writing – review & editing, Supervision. **Francesca Patrignani:** Conceptualization, Methodology, Validation, Resources, Writing – review & editing, Supervision, Project administration.

## Declaration of competing interest

The authors declare that they have no known competing financial interests or personal relationships that could have appeared to influence the work reported in this paper.

## Data Availability

All the data are available in the text
